# Markers of clinical and mitochondrial adaptation in response to moderate intensity continuous training: A systematic review and meta-analysis

**DOI:** 10.1371/journal.pone.0339902

**Published:** 2026-01-02

**Authors:** Veronica Vabishchevich, Ryan T. Smith, Adam J. Bittel

**Affiliations:** 1 Department of Physical Therapy, Rehabilitation, and Athletic Training, University of Kansas Medical Center, Kansas City, Kansas, United States of America; 2 Department of Physical Therapy, Utica University, Utica New York, United States of America; 3 Research Center for Genetic Medicine, Children’s National Hospital, Washington, District of Columbia, United States of America; Baskent University: Baskent Universitesi, TÜRKIYE

## Abstract

**Background:**

Aerobic exercise promotes mitochondrial morphological, enzymatic, and bioenergetic adaptions to improve muscle health and function. Although moderate intensity continuous training (MICT) is frequently recommended for sedentary and multiple clinical populations, there is little consensus regarding the effects of chronic MICT on these adaptations. The aim of this systematic review and meta-analysis is to evaluate the evidence for the effects of MICT on molecular transducers of mitochondrial biogenesis and cardiorespiratory fitness in adults.

**Methods:**

A comprehensive search was conducted in PubMed and CINAHL. Eligible studies assessed MICT lasting ≥2 weeks in adults, published since 2010, and collected vastus lateralis skeletal muscle biopsies pre and post chronic endurance exercise exposure. Data were extracted for mitochondrial transcription factor A (TFAM), citrate synthase (CS), peroxisome proliferator-activated receptor-gamma coactivator 1-alpha (PGC-1α), mitofusin 2 (MFN2), dynamin-related protein 1 (DRP1), VO₂max, and mitochondrial density (MitoVD). Meta-analyses using inverse-variance random effects models were conducted for outcomes reported in at least three studies.

**Results:**

A total of fourteen studies (n = 184) met inclusion criteria, with an overall low to moderate risk of bias and very low to low certainty of evidence. MICT significantly increased MitoVD (*p* < 0.00001) and VO₂max (*p* < 0.0001), while CS (*p* = 0.05) and MFN2 showed a modest increase (*p* = 0.01) following MICT. No changes were observed for TFAM, DRP1, or PGC-1α.

**Conclusion:**

MICT significantly improves MFN2 expression, CS activity, MitoVD, and VO_2_ max in adults. However, the overall quality of evidence is low. Heterogeneity in molecular responses suggests potential moderating effects of training duration, modality (e.g., cycling vs. treadmill), and sex – warranting further research.

**Registration:**

PROSPERO ID:CRD42024611640.

## 1. Introduction

Regular engagement in exercise and physical activity improves cardiorespiratory fitness, reduces the risk of developing type 2 diabetes mellitus (T2DM), obesity, and all-cause mortality [1–4]. In the United States it is estimated that 38.4 million adults have T2DM, 97.6 million individuals have prediabetes, and approximately 1.2 million adults will be newly diagnosed with T2DM each year [5]. Over the past two decades, there has been a substantial increase in obesity prevalence, affecting 40.3% of adults, and nearly 20% of children under 18 [6]. Current treatments for such cardiometabolic disorders typically involve education on lifestyle modifications (i.e., diet and exercise aimed at reducing weight and improving cardiorespiratory fitness), pharmacological management (e.g., incretin therapy), or in more severe cases, bariatric surgery [7]. The American College of Sports Medicine (ACSM) physical activity guidelines recommends adults participate in resistance training twice weekly, as well as 150 minutes of moderate-intensity exercise with a sustained heart rate range between 64–76% heart rate max, or 75 minutes of vigorous-intensity exercise at 77–95% heart rate max per week, or a combination of both [8]. Despite these well-established, and evidence-based recommendations, fewer than 25% of adults in the United States 18 or older meet the minimum physical activity guidelines [9].

Mitochondrial dysfunction and impaired cellular bioenergetics play a central role in the pathogenesis of chronic diseases such as diabetes and metabolic syndrome [1,10]. In physically trained individuals, mitochondria are elongated and enlarged, which increases mitochondrial density and improves bioenergetic efficiency [11,12]. These morphological adaptations are facilitated by altered mitochondrial enzyme activity, elevated mitochondrial protein synthesis, mitochondrial biogenesis, mitophagy to clear defective mitochondria, and mitochondrial fusion/fission dynamics [13,14]. In recent years, studies extensively examined effects of both chronic and acute mitochondrial adaptations to high intensity interval training (HIIT), as well as sprint-interval training (SIT), in both adults and athletes due to its time efficiency. [15–18]. However, the chronic adaptations to moderate intensity continuous training (MICT), a cornerstone of endurance exercise prescription that may more feasible and tolerable for sedentary or clinical populations, remain comparatively unexplored.

To our knowledge, no prior meta-analysis has synthesized the evidence across mitochondrial regulatory proteins, subsequent volumetric adaptations, and functional endpoints such as VO_2_ max in response to MICT. Because exercise promotes mitochondrial transcriptional, proteomic, and morphological adaptations that are critical to improved cardiorespiratory fitness and weight loss, understanding how MICT affects these pathways is essential for informing evidence-based exercise prescriptions in populations where high-intensity exercise may not be suitable. Therefore, this systematic review and meta-analysis aims to 1) evaluate the effect of MICT on key molecular transducers of mitochondrial biogenesis in human skeletal muscle, and 2) to assess whether these adaptations correspond to meaningful improvements in cardiorespiratory fitness in adults.

## 2. Methods

This systematic review protocol was registered through PROSPERO (ID: CRD42024611640) and conducted in accordance with PRISMA 2020 guidelines [19]. The three reviewers (VV, RTS, and AJB) determined inclusion and exclusion criteria prior to initiation of the database search and contributed to the entire systematic review process and subsequent meta-analysis. A systematic search was conducted using CINAHL Ultimate, and PubMed on 28 February 2024. Search terms and phrases were collectively composed and searched by the three reviewers, the search strategy is detailed in S1 Table. The search strategy involved each reviewer independently searching key and supplement terms for maximal phrase combination. For example, the key phrase “moderate intensity continuous exercise” was searched with supplemental terms “MitoVD” or “mitochondrial biogenesis” or “TFAM” or “PHF20” or “NRF1” or “PGC-1a”. Studies were included if they contained a MICT training group, assessed adults (>18), met moderate intensity parameters as outlined in Table 1, and utilized a pre-post measurement assessment with a vastus lateralis skeletal muscle biopsy for assessment of markers of mitochondrial biogenesis. No language restrictions were applied during the literature search.

**Table 1:** Defining exercise intensities.

Intensity	%VO_2_ max	%HR_peak_	%HR_reserve_	RPE	%Wpeak
Light	37-45	50-63	20-39	9-11	~50
Moderate	46-63	64-76	40-59	12-13	50-70
Vigorous	64-90	77-95	60-89	14-17	70-99
Maximal	≥91	≥95	≥90	≥18	100

Intensity criteria adapted from ACSM recommendations [20], Hansen et al. [21], and Vanhees et al. [22]. Metrics include percent maximal oxygen uptake: VO_2_ max, percent peak heart rate: %HR_peak_, percent heart rate reserve: %HR_reserve_, rate of perceived exertion: RPE, and peak power output: %W_peak_.

Studies that were considered for full-text review but were not written in English were reviewed by a reviewer fluent in the respective language. We excluded acute studies (single bout, or <2 weeks), studies assessing elite athletes, diet interventions, studies using non-continuous protocols, studies using protocols outside of moderate intensity, as well as studies assessing tissue other than skeletal muscle to increase generalizability and reduce the risk of confounding. A summary of the inclusion and exclusion criteria in Table 2. All eligible studies were combined into an independent file with duplicates removed. Title screening, abstract screening, and full article review was completed by three reviewers. For the full article review, articles were randomized, and two reviewers were randomly assigned for independent assessment. For an article to be included, both assigned reviewers were required to agree on their assessment independently. Any disagreements were discussed among all three reviewers until a final decision was reached.

**Table 2:** Study inclusion and exclusion criteria.

Inclusion	Exclusion
• Adults (≥18 years) • Specified moderate intensity intervention (must distinguish between HIIT, MICT, and SIT) • Skeletal muscle biopsy was performed for outcome assessment • Study design included pre- and post-intervention measurements • The intervention was described using FITT principles • Sample size ≥10 participants • A control condition was present, defined as no exercise or pre-exercise baseline	• Non-skeletal muscle tissue samples • Dietary interventions • Acute interventions (single-bout, < 2 weeks) • Enrolled elite athletes • Non-continuous exercise protocol • Moderate intensity protocol extended beyond the low- to moderate-intensity range (i.e., moderate-to-vigorous intensity)

Inclusion and exclusion criteria used to screen articles for systematic review and meta-analysis.

All available participant characteristics were extracted for participants including age, sex, and fitness levels. Existing baseline and follow-up anthropometric characteristics were extracted including weight, BMI, and VO_2_ max. Each reviewer extracted intervention data for frequency, duration, intensity, and mode as well as protein content relevant to mitochondrial biogenesis. We addressed outcome variability by pooling all relevant markers of mitochondrial biogenesis across studies. If three or more studies shared a relevant outcome, then it was included for synthesis. For any data that was unavailable within the manuscript or in the supplement, we contacted authors for raw data. For studies with unavailable raw data, a validated extraction software, WebPlotDigitizer (5.2), was used to extract data from provided graphs or synthesized a qualitative review [23,24]. For studies reporting data as mean and standard error of the mean (SEM), we converted these values to a standard deviation (SD) using the formula: $SD=SEM\times \;\sqrt{n}$, where n  is the sample size. The data used to complete the meta-analysis is available in the supporting material (S1 Data). Each reviewer completed a risk of bias assessment independently for each study using two assessment tools: Risk of Bias in Non-randomized Studies- of Intervention (ROBINS-I), and ‘Risk of Bias’ (RoB 2.0) for randomized intervention designs [25,26]. An automated form with prompts and signaling questions was used for both tools for accurate assessment of each bias domain.

Outcomes of protein prevalence, respiratory fitness, or mitochondrial density were treated as continuous values if collected at two time points: pre and post intervention. We calculated standard mean differences, and effect sizes (Cohen’s d) to detect any differences between the time points. Given the wide variety of outcomes related to mitochondrial biogenesis assessed in the systematic review, studies were grouped for meta-analysis: transcription factor A (TFAM), peroxisome proliferator-activated receptor gamma coactivator 1-alpha (PGC-1α), mitofusin 2 (MFN2), citrate synthase (CS), intermyofibrillar mitochondrial volume density (MitoVD%), dynamin-related protein 1 (Drp1) and VO_2_ max. There was variability in how protein content was reported, either as absolute amount, or fold-increase. For data reported as fold-increase, the corresponding author was contacted for access to the raw data. If the data was unavailable, the study was excluded from the meta-analysis. Using prior-established parameters to define moderate intensity, we were able to compare different interventions despite various reporting methods for exercise intensity. We conducted a random effects meta-analysis using the DerSimonian and Laird inverse-variance method to appropriately weight studies according to their sample size to assess the intervention effect. Heterogeneity among studies was evaluated using Cochrane’s Q statistics, and a Z-test was performed to determine the significance of the pooled effect estimate.

Statistical analysis and forest plot figure generation for the meta-analyses was completed in RevMan5 (Review Manager 5.4.1). Due to the limited number of studies available, and small sample sizes across the included studies, we did not perform sensitivity analyses for any outcome. To assess for small-study effects and publication bias, we initially intended to generate funnel plots to assess symmetry. However, our search yielded less than ten studies per meta-analysis, therefore we did not proceed with these analyses [27]. Finally, to evaluate the quality of the evidence, two reviewers independently assessed each meta-analysis in accordance with the GRADE handbook [28]. Any disagreement was resolved by a third reviewer.

## 3. Results

### 3.1. Participant characteristics

**Fig 1:**
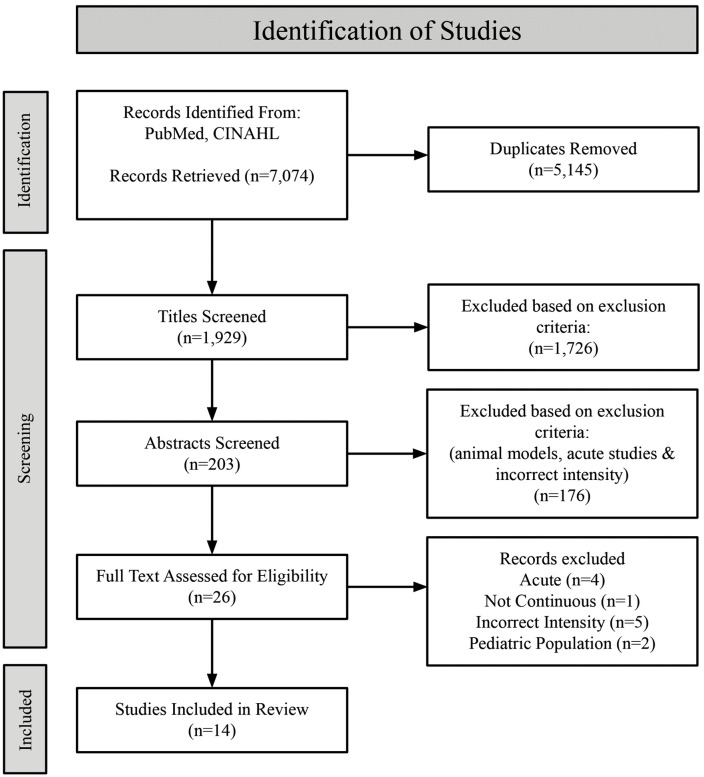
PRISMA flow diagram. The selection process for studies included in the systematic review and meta-analysis detailing the number of records identified, screened, and assessed for eligibility, as well as those included in the final synthesis.

A summary of the search strategy and rationale for exclusion is detailed in Fig 1. Studies that initially appeared to meet inclusion criteria but were excluded include those involving acute interventions [29–32], studies using non-continuous protocols [33,34], interventions with exercise parameters greater than moderate intensity (Table 1) [35,36], or studies involving pediatric populations [37,38]. A total of 14 studies completed across multiple countries met inclusion criteria for this systematic review and meta-analysis [39–52]. The pooled sample includes 184 predominately male participants, (Vaccari et al. [51] did not provide sex distribution for MICT group) with a pooled mean age range of 20–74 years (Table 3) [39–52].

**Table 3:** Summary of Included Studies.

Author	Participants	Intervention	Outcomes	Risk of Bias	Country
**Arribat et al. [39]**	Sedentary Older Adults Age: 66.2 ± 3.8 (11M/11F)	Bike/Walk/Run/Row 80% of training including walking or running at 3x/week for 16 weeks, 30-60 minutes at 75% HR_max_	MitoVD (%) DRP1 MFN2 VO_2_ Max	Low	Switzerland
**Broskey et al. [40]**	Sedentary Older Adults Age: 60–80 (7M/5F)	Bike/Walk/Run/Row 80% of training including walking or running at 3x/week for 16 weeks, 30-60 minutes at 75% HR_max_	MitoVD (%) TFAM PGC-1α VO_2_ Max	Moderate	Switzerland
**Gillen et al. [41]**	Sedentary Adults Age: 27 ± 8 (10M)	Cycling 3/week for 12 weeks For 45 minutes at 70% of HR_max_	CS VO_2_ Max	Low	Canada
**Granata et al. [42]**	Healthy Adults Age: 21 ± 2 (10M)	12 cycling sessions over 4 weeks exercising at 65% W_peak_	CS DRP1 MFN2 PGC-1α TFAM	Low	Australia
**Gunnarsson et al. [43]**	Healthy Adult Males Age: 25 (19–34) (7M)	Cycling 3 sessions/week for 8 weeks 60 minutes at ~60% VO_2max_	CS PGC-1α	Low	Denmark
**Konopka et al. [44]**	Healthy Adults Age: Young Cohort (20 ± 1) Age: Old Cohort (74 ± 3) (13M)	20–45 min for 3-4x/week for 12 weeks – 42 sessions (last 5 weeks at 80% HRR) 60-80% HRR cycling	CS PGC-1α VO_2_ Max MFN2	Low	United States
**Meinild Lundby et al. [45]**	Healthy Adults Age: 26 ± 4 (12M)	Cycling 4/week for 60 minutes for 6 weeks 65% W_peak_	CS MitoVD (%) MFN2 VO_2_ Max	Low	Switzerland
**MacInnis et al. [46]**	Healthy Adults Age: 23 ± 1 (10M)	Single leg cycling 6 sessions over 2 weeks at 50% W_peak_	CS MFN2	Low	Canada
**Montero et al. [47]**	Untrained healthy adults Age: 25 ± 4 years (16M)	Cycling 3–4/week for 60 minutes for 6 weeks at 65% W_peak_	MitoVD (%) VO_2_ Max	Moderate	Switzerland
**Ryan et al. [48]**	Sedentary Adults Age: 31 ± 6 (5M/10F)	4 Sessions/week of cycling/treadmill/ rowing/elliptical for 12 weeks at 70% HR_max_	VO_2_ Max	Moderate	United States
**Skelly et al. [49]**	Sedentary Adults Age: 28 ± 9 (10M)	Cycling 3/week for 12 weeks for 45 minutes with 2-week lead-in at 70% of max HR	MFN2	Low	Canada
**Skelly et al. [50]**	Healthy Young Adults Age: 21 ± 2 5M/5F	12 single leg cycling sessions over 4 weeks for 30 minutes exercising at ~50% W_peak_	CS MitoVD (%)	Low	Canada
**Vaccari et al. [51]**	Sedentary Obese Adults Age: 37.3 ± 0.6 (13 M/F)	Treadmill training for 3/week for 3 months at 60% VO_2_ peak 34 ± 0.14 sessions for 44 ± 8 min	CS	Low	Italy
**Youssef et al. [52]**	Sedentary Obese Adults Age: 68.1 ± 4.1 (18M/16F)	1 hr treadmill-based session 3x/week for 12 weeks 60-70% HR_max_ Borg 13–14	DRP1 TFAM MFN2	Low	Canada

Characteristics of study participants, including the number of males (M) and females (F), age (reported as range or mean ± standard deviation), outcomes extracted for meta-analysis, risk of bias, and country where study was conducted. Intervention characteristics are described by frequency, intensity, time, and type.

Of the 14 included studies only nine reported BMI, with averages ranging from 23–36.1 kg/m^2^ and studies by Youssef et al. [52] and Vaccari et al. [51] specifically recruited subjects with obesity [39–41,44,46,47,49,51,52]. Eight studies included sedentary adults but varied in defining sedentary behavior. [39–41,44,47,49,51,52]. For instance, four studies defined sedentary as either less than 2 hours of structured physical activity per week [52], less than 20 minutes of moderate continuous activity weekly as measured by the IPAQ-SF or self-report during initial medical screening [39,51], or as less than 600 MET-minutes of activity per week as measured by the IPAQ [41]. Conversely, several studies reported inclusion of untrained or inactive individuals without further detail [40,44,47,49]. Although the study by Meinild Lundby at al. [45] included participants who did not engage in a regular exercise, baseline assessments indicated that their cardiorespiratory fitness levels were higher than expected for sedentary adults.

Five studies recruited active adults; however, the definitions also varied across studies [42–44,46,49]. For example, Gunnarson et al. [43] included recreationally active adults, Konopka et al. [44] included active adults who exercised for more than 20 minutes/week in the last year, yet Granata et al. [42] defined moderately trained as no more than 3h/week of unstructured aerobic exercise. Lastly, two studies included participants who were habitually active but not engaged in sport-specific training [46,50]. Of note, none of the fourteen studies reported race or ethnicity.

### 3.2 Overview of included interventions

The included studies used various modes of aerobic exercise such as cycling, treadmill-based training, or a combination of aerobic-based exercises. Nine of the twelve included studies used only cycling based interventions spanning from two to 12 weeks [41–47,49,50]. Three of these cycling studies used a 12 week cycling intervention [41,44,49], one lasted 8 weeks [43], two studies used 6 week training protocols [45,47], two lasted 4 weeks [42,50], and only one study spanned two weeks [46]. The frequency of cycling interventions frequency mostly consisted of three to four sessions per week, ranging from 20–60 minutes, with variability in session length within each study (Table 3). For example, Konopka et al. [44] used a progressive protocol with an increase in intensity within the moderate to continuous range with session duration ranging from 20–45 minutes. The study by Granata et al. [42] was matched with a HIIT group for total work, and session duration was not reported. To establish exercise intensity, studies used measures of heart rate max (HR_max_), heart rate reserve (HRR), peak power output (W_peak_), or maximal oxygen intake (VO_2_ max). Five studies administered baseline maximal exercise testing to establish a W_peak_ threshold [42,45–47,50]. Two studies exercised participants at 50% W_peak_ [46,50], and 3 studies exercised participants at 65% W_peak_ [42,44,47]. In the studies by Skelly et al. [49] and Gillen et al. [41], participants cycled at 70% of HR_max_, while Konopka et al. [44] exercised participants at 60–80% of their HRR. Of the cycling studies, only Gunnarsson et al. [43] used VO_2_ max to monitor exercise intensity. Both Vaccari et al. [51] and Youssef et al. [52] implemented a treadmill-based exercise program, ranging from 48 minutes on average to an hour respectively. Using a 1-hour treadmill-based protocol, Youssef et al. [52] maintained an intensity of 60–70% of HR_max_ whereas the Vaccari et al. [51]maintained exercise intensity at 60% VO_2_ peak. Two studies used the same 16 week multi-modal aerobic intervention, allowing participants to select from biking, walking, running, or rowing, but ensured that 80% of the training time was either running or walking [39,40]. A complete summary of study, participant, and intervention characteristics is outlined in Table 3.

### 3.3. Risk of bias & GRADE

Risk of bias was assessed by the Risk of Bias in Non-Randomized Studies of Interventions (ROBINS-I), as well as the Risk of Bias Tool for Randomized Studies (RoB-2) [25,26]. Eight studies were assessed for bias using ROBINS-I [39,40,44,45,47–49,52]. We determined that three of these studies exhibited moderate bias due to issues in the selection of participants [48], and missing data domains [40,48]. Six studies were assessed for bias using ROB-2 [41–43,46,49,51]. Overall, we determined that these studies have a low risk of bias despite some concerns in the randomization domain. In this review, we primarily assessed physiologic and molecular adaptations that are unlikely to be influenced by randomization. Therefore, we did not feel that results from these studies would be overtly biased. Complete risk of bias assessment is outlined in the supplementary material (S2 Table and S3 Table). We recognize that skeletal muscle biopsies are gold-standard, and potentially invasive, resulting in lower sample sizes. However, the included studies encompass the highest quality of data currently available. Using the GRADE handbook, we determined that the overall certainty of evidence for each outcome is poor in quality due to small sample sizes, use of pre-post study design, as well as the lack of reporting of participant demographics (Table 4). Regarding VO_2_ max, the included studies used different normalizations for measures of VO_2_ max (if any), therefore we downgraded the certainty assessment for this outcome to very poor. Studies that assessed PGC-1α used either western blotting to assess for protein expression, or mRNA expression, which may not always reflect true protein content. Because of this, we downgraded the certainty of evidence to very low due to imprecision. Similarly, different methods were used to determine mitochondrial density, therefore we downgraded the certainty of the evidence for this outcome to very low. Overall, the pooled effect for CS was not significant, with contrasting outcomes, therefore due this imprecision, we downgraded the certainty of evidence for this outcome to very low. Similarly, the pooled effect for DRP1 was not significant, with studies trending in different directions post-intervention. Therefore due to this imprecision, we graded the certainty of evidence DRP1 as very low.

**Table 4:** Summary of GRADE assessment for the quality of evidence across outcomes.

Quality Assessment							No. of Observations		Effect	Quality
No. of studies	Study Design	Risk of Bias		Inconsistency	Indirectness	Imprecision	Other Considerations	Pre	Post	Standard Mean Difference (95% CI)
**Mitochondrial Volume Density** Arribat et al. [39], Broskey et al. [40], Meinild Lundby et al. [45], Montero et al. [47], and Skelly et al. [49]										
5	Pre-Post	Not serious	Not serious	Not serious	Serious	None	48	48	1.04 [0.68, 1.40]	⊕○○○ Very Low
**Citrate Synthase Activity** Gillen et al. [41], Granata et al. [42], Gunnarsson et al. [43], Meinild Lundby et al. [45], MacInnis et al. [46], Skelly et al. [50], and Vaccari et al. [51]										
7	Pre-Post	Not serious	Not serious	Not serious	Serious	None	58	58	0.48 [−0.00, 0.96]	⊕○○○ Very Low
**VO**_**2**_ **Max** Arribat et al. [39], Broskey et al. [40], Gillen et al. [41], Konopka et al. [44], Meinild Lundby et al. [45], Montero et al. [47], & Ryan et al. [48]										
7	Pre-Post	Not serious	Serious	Not serious	Not Serious	None	67	72	0.75 [0.37, 1.14]	⊕○○○ Very Low
**Mitofusin 2** Arribat et al. [39], Granata et al. [42], Meinild Lundby et al. [45], MacInnis et al. [46], Skelly et al. [50], and Youssef et al. [52]										
6	Pre-Post	Not serious	Not serious	Not serious	Not Serious	None	45	45	0.40 [0.08, 0.73]	⊕⊕○○ Low
**PGC-1α** Broskey et al. [40], Granata et al. [42], & Gunnarsson et al. [43],										
3	Pre-Post	Not serious	Not serious	Not serious	Serious	None	23	23	0.50 [−0.09, 1.09]	⊕⊕○○ Low
**TFAM** Broskey et al. [40], Granata et al. [42], and Youssef et al. [52]										
3	Pre-Post	Not serious	Not serious	Not serious	Not Serious	None	31	31	0.45 [−0.06, 0.96]	⊕⊕○○ Low
**DRP1** Arribat et al. [39], Granata et al. [42], Youssef et al. [52]										
3	Pre-Post	Not serious	Not serious	Not serious	Serious	None	45	42	0.26 [−0.16, 0.69]	⊕○○○ Very Low

The domains were rated as no concern, some concern, or serious concern for each respective quality domain. Each outcome effect size was reported as the standard mean difference with a 95% confidence interval. Overall study quality was rated with the following scale: very low (⊕), low (⊕⊕), moderate (⊕⊕⊕), or high (⊕⊕⊕⊕).

### 3.4. Mitochondrial volume density

**Fig 2:**
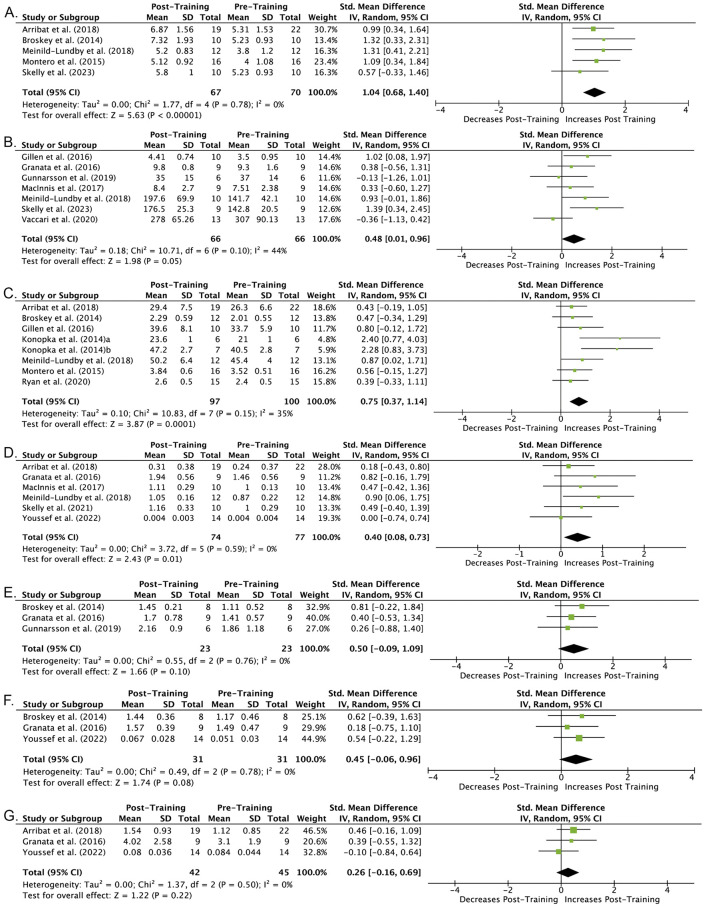
Forest plots of meta-analyses for markers relating to mitochondrial biogenesis. Effect of moderate intensity continuous training on: (A) Mitochondrial Volume Density, (B) Citrate Synthase Activity, (C) VO_2_ max, D) Protein Content of MFN2, E) PGC-1α, (F) TFAM (G) DRP1. Positive values favor an increase at post-training, and negative values favor decrease/no change at post-training. Effect sizes are expressed as standardized mean differences (SMD) with 95% confidence internals. Heterogeneity was assessed using the I^2^ statistic. a* young cohort, b* older cohort.

Five included studies estimate the effect of MICT on mitochondrial volume density (MitoVD) [39,40,45,47,50]. Three of these studies included healthy middle-aged subjects [45,47,50], while two studies included older males and females ages 60–80 years. [39,40] Similarly, while Skelly et al. [50], Meinild Lundby et al. [45] and Montero et al. [47] conducted interventions ranging from 4–6 weeks, studies by Broskey et al. [40] and Arribat et al. [39] conducted similar 16-week supervised MICT programs. We included all five of these studies in an inverse-variance meta-analysis. Fig 2A presents a forest plot with effect sizes (Cohen’s d) of the change in skeletal muscle mitochondrial volume density pre- and post-MICT for each study, study sample sizes, and the pooled weighted effect size. Overall, the effects sizes for MICT were large on MitoVD, ranging from 0.59 to 1.63, with only low heterogeneity between studies (I^2^ = 0%, Cochrane’s Q: p = 0.78). We found a significant pooled effect estimate of 1.04 [95% CI: 0.68, 1.40] (z = 5.63, p < 0.00001), confirming that MICT is associated with elevated mitochondrial volume density.

### 3.5. Citrate synthase activity

Citrate synthase activity is commonly used as a biomarker of mitochondrial content in skeletal muscle due to its positive correlation with measures of mitochondrial DNA content [53]. Eight studies included estimates of the effect of MICT on citrate synthase (CS) activity [41–46,50,51]. The study by Gillen et al. [41] found equivalent improvements in CS between shorter/high-intensity, and longer/moderate-intensity exercise regimens, while MacInnis et al. [46] reported superior improvements in CS activity following HIIT (65% average W_peak_) than MICT (50% W_peak_). The study by Granata et al. [42] reported similar findings, with only sprint interval training (30 second bouts at ~200% W_peak_), resulting in improved mitochondrial-specific respiration normalized to CS activity. Likewise, Konopka et al. [44] found increases in both young and old participants after 12 weeks of cycling at 60−80% HRR, however the data were reported as a percent increase. In contrast, the study by Skelly et al. [50] reported significant increases in CS activity in continuous, but not interval, training performed at the same intensity (50% W_peak_). Additionally, the study by Meinild Lundby et al. [45] also found significant increases in CS activity (by 44% in healthy men in response to 6 weeks of training). However, as indicated above, not all studies observed significant increases in citrate synthase activity after MICT. Granata et al. [42] reported small but non-significant increases in CS activity with standardized effect size of 0.38, while the study by Gunnarsson et al. [43] reported a small decrease in CS activity following 60 min of moderate intensity continuous cycling at 157 ± 20 W with or without inclusion of 30 second sprints (standardized effect size of −0.13). Similarly, Vaccari et al. [51] found no significant change in CS activity in response to 3 months of MICT at 60% VO_2_ peak or HIIT at 100% VO_2_ peak (standardized effect size of −0.47). We included seven studies (Konopka et al., [44] unable to get raw data) in an inverse-variance meta-analysis. Fig 2B presents a forest plot with effect sizes (Cohen’s d) of the change in CS activity pre- and post-MICT for each study, study sample sizes, and the pooled weighted effect size. Overall, we observed moderate heterogeneity between studies (I^2^ = 44%, Cochrane’s Q: p = 0.10). We found a non-significant pooled effect estimate of 0.48 [95% CI: 0.01, 0.96] (z = 1.98, p = 0.05) of MICT on CS activity.

### 3.6. VO_2_ Max

Maximal oxygen uptake (VO_2_ max) is a critical measure of cardiorespiratory fitness influenced by several factors, including pulmonary diffusion capacity, cardiac output, oxygen carrying capacity of blood, and skeletal muscle characteristics such as the expression of mitochondrial enzymes, mitochondrial content, and capillary density [54]. Seven studies included estimates of the effect of MICT on VO_2_ max [39–41,44,45,47,48]. All seven reported statistically significant improvements in VO_2_ max following MICT, with the percent increases ranging from 9% [47] to 19% [41] across training periods lasting from 6 weeks [45,47] to 16 weeks [39,40]. Of these, four studies included only young male subjects [41,44,45,47], three studies included older males [39,40,44], and only three studies included women [39,40,48]. For accurate comparison of cardiopulmonary fitness across individuals, measures of VO_2_ max should be normalized to lean mass as opposed to body weight, as fitness can be underestimated with higher body fat content (e.g., in individuals with obesity). [55,56] The study by Konopka et al. [44] recruited a young (a) and older (b) cohort, and are listed separately in Fig 2C. Of these five studies, only Broskey et al. [40] normalized VO_2_ max to lean body mass. Of the remaining studies, four normalized to body weight [41,44,45], while Montero et al. [47] reported VO_2_ max using un-normalized units of L/min. We included all seven studies in an inverse-variance meta-analysis. Fig 2C presents a forest plot with effect sizes (Cohen’s d) of the change in VO_2_max pre- and post-MICT for each study, study sample sizes, and the pooled weighted effect size. Overall, we observed low heterogeneity between studies (I^2^ = 35%, Cochrane’s Q: p = 0.15). We found a significant pooled effect estimate of 0.75 [95% CI: 0.37, 1.14] (z = 3.87, p < 0.0001) of MICT on VO_2_max.

### 3.7. Mitofusin 2

Mitofusin 2 (MFN2) is a dynamin-like GTPase membrane protein that promotes mitochondrial fusion – a critical process in mitochondrial quality control that facilitates the exchange of lipid membranes and proteins between mitochondria, promotes mitochondrial repair, and enables removal of defective mitochondria [57]. Seven studies evaluated the effect of MICT on MFN2 protein content. [39,42,44–46,49,52] Five of the studies reported significant increases in MFN2 content [39,42,44,49,52], however studies by Youssef et al. [52], and MacInnis et al. [46] did not find any significant difference. The conflicting results may be due to several reasons. Regarding sex, Youssef et al. [52] and Arribat et al. [39] included women, however the other five studies only enrolled male participants. [42,44,45,49,52] Additionally, both Youseff et al. [52] and Arribat et al. [39] assessed both older men and women, while the other three studies assessed young men. [42,45,49] The length of intervention was also variable across studies ranging from 2 to 16 weeks suggesting duration of training affects the MFN2 response to MICT. For instance, Arribat et al. [39] conducted a 16-week training period, Youssef et al. [52] and Konopka et al. [44] utilized a 12-week training period, Granata et al. [42], and Meinild Lundby et al. [45] used 6-week intervention periods, and MacInnis et al. [46] used a two week intervention period. Lastly, exercise mode may influence the pattern of MFN2 expression. Both Youssef et al. [52] and Skelly et al. [49], used similar training frequencies (3x/wk) and intensities (65−70% HR max) but different exercise modalities (treadmill in study by Youssef et al. [52] versus cycling in the studies by Skelly et al. [49]), which may contribute to the different effects of MICT. We included six of these studies in an inverse-variance meta-analysis (Konopka et al. [44] excluded due to inability to get raw data). Fig 2D presents a forest plot with effect sizes (Cohen’s d) of the change in MFN2 protein content pre- and post-MICT for each study, study sample sizes, and the pooled weighted effect size. Overall, we observed low heterogeneity between studies (I^2^ = 0%, Cochrane’s Q: p = 0.59). We found a significant pooled effect estimate of 0.40 [95% CI: 0.08–0.73] (z = 2.43, p = 0.01) of MICT on MFN2 protein expression.

### 3.8. PGC-1α

PGC-1α is a transcription co-activator that regulates several transcription factors involved in adaptive thermogenesis, mitochondrial biogenesis, glucose and fatty acid metabolism, fiber-type switching to oxidative phenotypes, and cardiac development [58]. Four studies evaluated the effect of MICT on skeletal muscle PGC-1α mRNA or protein content in exclusively male participants [40,42–44]. The MICT programs lasted from 4 to 16 weeks by Broskey et al. [40], and Granata et al. [42], respectively and consisted almost exclusively of cycling exercise. Two studies reported measures of PGC-1α mRNA [40,43], while two studies reported changes in PGC-1α protein content [42,44]. Three of these studies were included in an inverse-variance meta-analysis (Konopka et al. [44] could not get raw data). [40,42,43] Fig 2E presents a forest plot with effect sizes (Cohen’s d) of the change in PGC-1α mRNA or protein expression pre- and post-MICT for each study, study sample sizes, and the pooled weighted effect size. Overall, we observed low heterogeneity between studies (I^2^ = 0%, Cochrane’s Q: 0.50, p = 0.76). We found a non-significant pooled effect estimate of 0.51 [95% CI: −0.09, 1.09] (z = 1.66, p = 0.10) of MICT on PGC-1α expression.

### 3.9. TFAM

Mitochondrial Transcription Factor A (TFAM) is a nuclear encoded gene that regulates mitochondrial transcription and maintenance, for example, by binding to the promoter region of mitochondrial DNA and maintains recognition sites for NRF1 and NRF2, which regulate the transcription of mitochondrial genes encoding electron transport chain complexes 1−5, mitochondrial membrane transporters, mitochondrial ribosomes, and multiple mitochondrial transcription factors that support biogenesis [59]. Three studies included an assessment of the effect of MICT on TFAM gene and/or protein expression. [40,42,52] Two of these studies reported increases in TFAM protein expression [40,52]; Granata et al. [42] investigated the effect of MICT on TFAM mRNA expression. Of note, both Youssef et al. [52], and Broskey et al. [40], reported significant increases in TFAM protein and mRNA expression, respectively, while Granata et al. [42] failed to identify changes in protein content in response to MICT. These differences could be due to differences in training duration (12−16 weeks vs. 4 weeks), or the younger age of participants in the study by Granata and colleagues [42]. All three studies were included in an inverse-variance meta-analysis. Fig 2F presents a forest plot with effect sizes (Cohen’s d) of the change in TFAM mRNA or protein expression pre- and post-MICT for each study, study sample sizes, and the pooled weighted effect size. Overall, we observed low heterogeneity between studies (I^2^ = 0%, Cochrane’s Q: p = 0.78). We found a non-significant pooled effect estimate of 0.45 [95% CI: −0.06, 0.96] (z = 1.74, p = 0.08) of MICT on TFAM expression.

### 3.10. DRP1

Dynamin-related protein (DRP1) is a GTPase that is recruited to the mitochondria, where it constricts and cleaves the mitochondrion to facilitate fission [60]. Three studies included an assessment of the effect of MICT on DRP1 expression [39,42,52]. The study by Youssef et al. [52] failed to find a significant change in the protein content of DRP1 in older men and women performing one hour of treadmill-based MICT at 60−70% maximal heart rate 3 times per week for 12-weeks in the vastus lateralis. The study by Granata et al. [42] found only a trend toward increased DRP1 protein content in the vastus lateralis in younger men in response to 4 weeks of cycling MICT performed 3x/wk at 90–97.5% of work at the lactate threshold. Lastly, Arribat et al. [39] using a multi-modal aerobic intervention lasting 16 weeks also observed a non-significant increase DRP1 content. All three studies were included in an inverse-variance meta-analysis. Fig 2G presents a forest plot with effect sizes (Cohen’s d) of the change in DRP1 protein content pre- and post-MICT for each study, study sample sizes, and the pooled weighted effect size. All three studies were included in an inverse-variance meta-analysis (Fig 2G). Overall, there is low heterogeneity between studies (I^2^ = 0%, Cochrane’s Q: 1.37, p = 0.5). We found a non-significant pooled effect estimate of 0.26 [95% CI: −0.16, 0.69] (z = 1.22, p = 0.22) for the effect of MICT on DRP1 content.

## 4. Discussion

### 4.1. Mitochondrial biogenesis as an adaptive response

**Fig 3:**
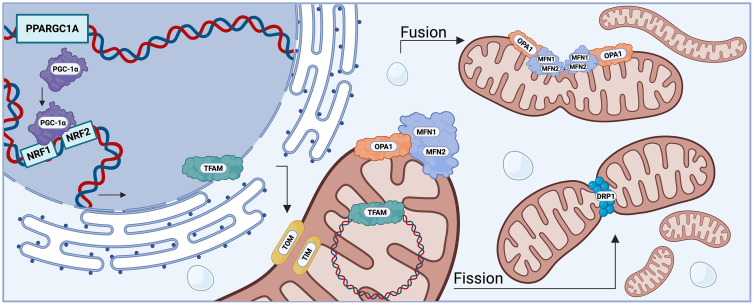
Mitochondrial biogenesis and dynamics in human skeletal muscle in response to exercise. Endurance type exercise increases expression of *PPARGC1A* (PGC-1α) which leads to an elevated level of PGC-1α. This coactivator interacts with transcription factors NRF1 and NRF2 promoting expression of TFAM, a protein integral for the initiation of mitochondrial biogenesis. TFAM is synthesized in the cytosol and imported into the mitochondrial matrix via TOM (Translocase of the Outer Membrane) and TIM (Translocase of the Inner membrane) complexes. Inside the mitochondria, TFAM binds to mtDNA to regulate replication and transcription. Mitochondrial morphology is maintained through dynamic remodeling: fusion is mediated by MFN1/MFN2 (outer membrane) and OPA1 (inner membrane), while fission is directed by the cytosolic protein DRP1 is recruited to the mitochondrial surface where is assembles into a ring-like structure, constricting and dividing the organelle into two distinct mitochondria. Together, these pathways ensure mitochondrial quality and adaptability to the energetic demands imposed by exercise in skeletal muscle cells. Figure generated in BioRender.com [68].

This systematic review and meta-analysis synthesized the current evidence regarding the effect of MICT on the expression of key molecular transducers of mitochondrial remodeling (PGC-1α, TFAM, DRP1, MFN2), as well as citrate synthase activity, mitochondrial volume density, and cardiorespiratory adaptations (VO_2_ max) traditionally associated with aerobic exercise training. While improvements in cardiorespiratory fitness are multifactorial, the skeletal muscle determinants of oxygen delivery and utilization by mitochondria include myofiber cross-sectional area, fiber type composition, muscle fiber capillarization, and skeletal muscle mitochondrial density [61]. Indeed, skeletal muscle has multiple molecular mechanisms to tune mitochondrial respiration and mitochondrial content to current energy demands. To maximize oxidative capacity, mitochondria can fuse (fusion) to facilitate the sharing of proteins, lipids, and RNA in a process that relies on expression of mitofusion-1 (MFN1) and MFN2 (regulating outer mitochondrial fusion), and OPA1 (regulating inner membrane fusion) [62]. As a quality control mechanism, mitochondria can also divide (fission) to help populate growing or dividing cells with a sufficient number of mitochondria or facilitate mitophagic removal of defective mitochondria in a process that relies on DRP1 [63]. Additionally, exercise training is associated with upregulation of the transcriptional co-activator PGC-1α, a key regulator of mitochondrial biogenesis through its interaction with NRF1 and NRF2, and subsequent upregulation of mitochondrial transcription factor A (TFAM) that promotes formation of new mitochondria to support respiration [64,65]. Additionally, NRF2 transcription has also been shown to increase in response to exercise-mediated oxidative stress, as a means of restoring redox balance. However, the role of NRF2 in mitochondrial biogenesis is equivocal -- some studies reporting no loss of biogenesis in response to NRF2 knockdown [66], while others report deficits in biogenesis [67]. An overview of mitochondrial biogenesis is detailed in Fig 3.

The findings from the meta-analysis suggest that MICT promotes improvements in MFN2 expression, citrate synthase activity (biomarker of mitochondrial content in skeletal muscle) (p = 0.05), mitochondrial volume density, and VO_2_ max, which may have important implications for metabolic health and muscle function, specifically in the vastus lateralis. Because MICT depends on aerobic respiration, increasing training in this range may facilitate favorable mitochondrial adaptations [69]. However, while the studies in this review maintained a low risk of bias (with some exceptions for bias in the selection of participants, missing data, and randomization) across the domains of ROBINS-I and ROB-2, the quality of evidence was rated as low. Therefore, while we found significant, and at times large, pooled effect estimates for some outcomes, these results should be interpreted cautiously. Further research is needed to strengthen the certainty of the effect of MICT on the molecular transducers of mitochondrial adaptation in human skeletal muscle.

### 4.2. PGC-1α and MICT

Our meta-analysis of the results of MICT on PGC-1α expression generally supported no change with training, which coincides with the lack of an effect on TFAM content (a downstream transcription factor induced by PGC-1α expression) [70]. Previous research indicates that PGC-1α mRNA expression increases in an intensity-dependent manner when controlling for total energy expenditure and that different PGC-1α isoforms may respond differently to altered training intensities (e.g., PGC-1α B, which increases in response to lower-intensity training) [71]. Therefore, MICT may not be intense enough to stimulate PGC-1α expression, or may stimulate other isoforms of this transcriptional co-activator not measured in the studies reviewed here. The kinetics of PGC-1α may also account for these findings, as muscle biopsies may have been taken after or before transcriptional/translational changes in expression [72]. These results further suggest that increased mitochondrial volume density may occur primarily through an increase in mitochondrial size (e.g., mitochondrial enlargement) or an increase in length density rather than through biogenesis (see discussion of the effects of MICT on intermyofibrillar MitoVD below) [73]. Further research is needed to delineate the effects of exercise intensity and duration on PGC-1α isoforms to further understand mitochondrial adaptations.

### 4.3. MFN2 and MICT

Increased expression of MitoVD, VO_2_ max, and CS activity could be related in part to the significant increases in MFN2 (pro-fusion) and no change in DRP1 (pro-fission) expression. Previous studies have reported increased mitochondrial volume in response to aerobic exercise training secondary to mitochondrial enlargement, which has been associated with increased VO_2_ max [74]. In contrast, mitochondrial fragmentation has been linked to skeletal muscle inflammation, which precedes muscle atrophy and functional impairment – suggesting that proteomic or transcriptional changes pushing mitochondria toward fusion may be critical to improve muscle function in response to exercise [75].

Mitochondria undergo fusion, fission, and autophagy to meet the metabolic demands in skeletal muscle. Mitochondrial fusion, partly regulated by MFN2 and its dimerization partner, MFN1 undergoes GTP hydrolysis triggering conformational changes that initiate fusion of the outer membrane of separate mitochondrion, forming denser and dynamic mitochondrial networks [74,76–78]. In this review, studies evaluating MFN2 showed different results which could be partly explained by different characteristics among participants. For instance, Youssef et al. [52], who did not identify changes in MFN2 expression,included both male and female older adults, while Granata et al. [42], Meinild Lundby et al. [45] and Skelly et al. [49], who all found significant effects of MICT on MFN2 expression, included healthy young men. Thus, the discrepancy in results by Youssef et al. [52] may be due to a longer intervention time, participant age, or the inclusion of female participants, or a combination of all three. Our findings suggest that in response to chronic MICT there may be an effective increase in MFN2 protein content. MFN2 presence and function is critical to ensure secure tethering of the mitochondria to the endoplasmic reticulum allowing Ca^2+^ transfer for cellular signaling, mitochondrial metabolism, and ATP production [79]. In mouse models, a decline in MFN2 expression in skeletal muscle has been linked to age-related sarcopenia.[80]. However, the effect of sex on responses to MICT are less clear, with one study suggesting no sex-based differences in MFN2 protein content [81]. Additionally, suppression of MFN2 in skeletal muscle from patients with obesity and T2DM has been associated with the development of insulin resistance [79]. Work by Bell et al. [82] suggests that knockout of MFN1 or MFN2 individually does not change to time to exhaustion or exercise performance, but individual knockout of MFN2 results in a steep decline in exercise performance, further suggesting that MFN2 expression is linked to endurance, and cardiorespiratory fitness. Given its role in exercise endurance and performance, further research is warranted to clarify and expand upon these findings.

### 4.4. Intermyofibrillar MitoVD and MICT

Although all four included studies assessing for changes in intermyofibrillar mitochondrial volume density had variability in participant age and training duration, they all reported increases in MitoVD in response to MICT, resulting in a highly significant pooled effect estimate (Fig 2A). However, the increase reported by Meinild Lundby et al. [45], occurred without concomitant increases in mitochondrial content (number), further indicating that MICT may have increased mitochondrial volume alone. Assessment of mitochondrial volume vs. mitochondrial number is an important distinction not made in the studies by Arribat et al. [39], Skelly et al. [50], Montero et al. [47], or Broskey et al. [40], who used grid-based, point-counting stereology approaches. Therefore, in these studies it is difficult to determine if elevations in mitochondrial density were due to an increase in mitochondrial biogenesis, or volume, or both. Overall, the effect of aerobic exercise on mitochondrial volume have been equivocal, with some studies reporting an increase in volume density [83–87], while studies by Zoladz et al. [88], and Botella et al. [89], report declines or no change. Likewise, Nielsen et al. [90] also reported non-volumetric adaptations, including increases in mitochondrial cristae density, which was shown to be a better predictor of cardiorespiratory fitness than mitochondrial volume (but again, this study did not report changes in the absolute number of mitochondria). Likewise, the recent study by Botella et al. [89] reported increased mitochondrial cristae density, decreased mitochondrial size, and an increase in the surface-area-to-volume ratio of mitochondria from strength-trained athletes without a change in mitochondrial network volume – indicating an increase in mitochondrial number (more mitochondria of smaller size occupying the same volume). Given the importance of mitochondrial morphology to respiratory function and cardiorespiratory fitness, further research is needed to determine if MICT provides superior increase mitochondrial volume compared to HIIT, with specific distinction between cristae volume, mitochondrial volume (per mitochondria), and number of mitochondria to more carefully delineate the observations observed.

### 4.5. VO_2_ max and MICT

Maximal oxygen uptake (VO_2_ max) during whole-body aerobic exercise is primarily limited by the capacity of the cardiorespiratory system to deliver oxygen to skeletal muscle [54]. Although increases in mitochondrial enzymes are frequently observed following aerobic exercise, these adaptations alone are unlikely to be the primary drivers of enhanced VO_2_ max. Previous research indicates that increases in VO_2_ are modest despite substantial increases in mitochondrial volume, therefore the relationship between enzymatic mitochondrial content adaptation is not directionally proportional [54]. However, a strong correlation does exist between relative VO_2_ max (l/min^-1^/kg^-1^) and mitochondrial volume density, but not necessarily absolute VO_2_ (l/min^-1^) [91]. Chronic exercise training induces hematologic adaptations, including hypervolemia [92]. To control for these adaptations, Montero et al. [47], through phlebotomy removed the excess blood volume following training. While their findings demonstrate that cardiac output, red blood cell volume, plasma volume, and hemoglobin mass remained as independent predictors of VO_2_ peak, MitoVD or inter-myofibrillar MitoVD was not. With a small sample size of 16 participants and considerable variability in body mass, VO_2_ peak may be overestimated given the lack of normalization for body mass [47]. Previous studies have demonstrated that for every 1 MET (3.5 mL.min^-1^/kg^-1^) improvement in exercise capacity in adults there is a 12–15% reduction in mortality [93,94]. Following endurance training ranging from 6–16 weeks, the younger cohort from Konopka et al. [44], Broskey et al. [40] (normalized VO_2_ to lean mass), Gillen et al. [41], and Meinild Lundby et al. [45] each met or exceeded this 1 MET threshold. These findings provide further support that MICT even over short durations can produce clinically meaningful improvements in aerobic fitness.

### 4.6. Clinical implications of MICT

Within skeletal muscle there is a hierarchical order of muscle fiber recruitment with low to moderate intensity endurance exercise initially stimulating highly aerobic type I muscle fibers, with endurance athletes exhibiting a greater proportion of type I muscle fibers [95,96]. Interestingly, individuals with T2DM tend to exhibit a shift from slow-twitch (type I) fibers to a greater proportion of fast twitch-fibers [75,76]. As type I fibers have a higher mitochondrial density as compared to other fiber types; stimulation of these fiber types may be a target of therapeutic exercise inducing hypertrophy resulting in a greater cross-sectional area [97]. Previous research indicates that increased mitochondrial content in human skeletal muscle are associated with improved cardiorespiratory performance and fitness, which represents a major goal of prescribing exercise regimens in these populations [78,90,98].

Current research has maintained an intense focus on the utility of high intensity interval training (HIIT) as a treatment for metabolic and inherited muscle diseases. A recent systematic review and meta-analysis addressing compliance and adherence rates between HIIT and MICT to be comparable, however the overall quality of the evidence was poor [99]. Likewise, another review assessed the long-term adherence between MICT and HIIT, finding that over long term, individuals prescribed to HIIT generally exercised at lower than prescribed intensities, with no advantage to long term adherence [100]. As MICT can usually be performed without supervision, it serves as an attractive introductory option for individuals who are untrained, and who would not tolerate or adhere to sustained elevated heart rate levels. Leveraging the mechanisms driving mitochondrial biogenesis, commitment to a MICT exercise regimen may support mitochondrial function in individuals who are untrained and sedentary. However further investigation in this area is warranted. Given the rise of clinical conditions like prediabetes and T2DM, there is a need for further research assessing the effect of exercise modalities on mitochondrial dynamics, and its effects on glucose homeostasis in the development of personalized and effective intervention strategies mitigating chronic disease progression.

## 5. Strengths & limitations

To our knowledge, this is the first systematic review and meta-analysis assessing measures of mitochondrial biogenesis in response to chronic moderate intensity aerobic training. We identified substantial heterogeneity in the reporting of exercise intensity in previous studies; however, we applied established intensity thresholds (Table 1) to classify training interventions as moderate intensity, facilitating comparability across studies with diverse protocols. Importantly, studies in this review utilized skeletal muscle biopsies, enabling direct measurement of mitochondrial density and accurate assessment of skeletal muscle protein content. Despite these strengths, several limitations warrant consideration. First, we did not extract data from a sedentary control group, as most studies implemented a HIIT or sprint-interval training comparator, which is beyond the scope of this review. Instead, we analyzed pre-post data, allowing participants to serve as their own controls, thereby reducing within-subject variability. Second, participant demographics were often incompletely reported, specifically regarding race and ethnicity, which limits the generalizability of our findings. Finally, although muscle biopsies are the gold-standard for assessing mitochondrial adaptations, their invasive nature likely contributed to small sample sizes observed across studies. Myofiber subtypes also demonstrate unique metabolic, contractile, and transcriptional responses to exercise training – further confounding the relationship between MICT and mitochondrial adaptation [101].

## 6. Conclusion

This meta-analysis provides evidence that MICT leads to meaningful mitochondrial adaptations including increased mitochondrial volume (MitoVD), upregulation of key regulatory factors in mitochondrial fusion (e.g., MFN2), and increased citrate synthase activity. Evidence further supports that MICT induces clinically meaningful improvements in cardiorespiratory fitness VO_2_ max – underscoring the physiologic relevance of these adaptations. Taken together, MICT may constitute a viable and accessible strategy for enhancing metabolic health, particularly in populations that cannot tolerate vigorous exercise. However, relatively few studies were available for the included outcomes in this review, and the quality of available evidence is low due to the use of small sample sizes, and inconsistent measurement approaches. Future research is needed to strengthen the certainty of these findings and should assess the extent and temporal nature of mitochondrial adaptations, including the effects of longer-term follow-up. Additionally, investigations in clinical populations, such as those with prediabetes, T2DM, or metabolic syndrome are warranted to better inform personalized exercise prescription.

## Supporting information

S1 Table Search Strategy. Search strategy used for systematic review and meta-analysis, including databases searched, key phrases, supplemental terms, and example search query. Searches were conducted as detailed in the methods section of the main manuscript. Outlined is the full search used in PubMed; a similar strategy was adapted for CINAHL Ultimate.
(DOCX)

S2 Table Risk of Bias (ROBINS-I). Risk of Bias assessment for non-randomized studies of interventions (ROBINS-I).
(DOCX)

S3 Table Risk of Bias (ROB-2). Risk of Bias assessment for randomized trials (ROB-2).
(DOCX)

S1 Data Meta-Analysis. (XLSX)

## Abbreviations

CS: Citrate Synthase
DRP1: Dynamin-Related Protein 1
FITT: Frequency, Intensity, Time, Type
HIIT: High Intensity Interval Training
HRR: Heart Rate Reserve
HR_max_: Maximal Heart Rate
MFN1: Mitofusin 1
MFN2: Mitofusin 2
MICT: Moderate intensity continuous training
MitoVD: Mitochondrial volume density
NRF1: Nuclear Respiratory factor 1
NRF2: Nuclear Respiratory factor 2
OPA1: Optic atrophy 1 gene
%VO_2_ max: Percent maximal oxygen uptake
%HR_peak_: Percent peak heart rate
%HR_reserve_: Percent heart rate reserve
PGC-1α: Peroxisome proliferator-activated receptor-gamma coactivator 1-alpha
PHF20: Plant homeodomain finger-containing protein 20
RPE: Rate of Perceived Exertion
SIT: Sprint interval training
TFAM: Mitochondrial transcription factor A
T2DM: Type 2 Diabetes Mellitus
W_peak_: Peak power output
