# Supplementary material for: Markers of clinical and mitochondrial adaptation in response to moderate intensity continuous training: A systematic review and meta-analysis
Source: PLoS One. 2026 Jan 2;21(1):e0339902. doi: 10.1371/journal.pone.0339902 (PMC12758752; doi:10.1371/journal.pone.0339902)
Supplement: S1 Table — Search strategy used for systematic review and meta-analysis, including databases searched, key phrases, supplemental terms, and example search query. Searches were conducted as detailed in the methods section of the main manuscript. Outlined is the full search used in PubMed; a similar strategy was adapted for CINAHL Ultimate. (DOCX) [file pone.0339902.s001.docx]

**Supplemental Table 1.** **Search Strategy**

Databases: PubMed, and CINAHL Ultimate

Key Phrases:

“Moderate intensity continuous exercise”, “MICT”, “Continuous Aerobic Exercise”, “Moderate intensity exercise”, “skeletal muscle”, “aerobic exercise”, “sub-lactate threshold continuous training”, “physical activity”, “endurance training”, “exercise”, and “walking”.

Supplemental terms:

“MitoVD”, “mitochondrial biogenesis”, “TFAM”, “PHF20”, “NRF1”, and “PGC-1a”.

Example)

All articles returned from two databases from the following phrases: “moderate intensity continuous exercise” and “MitoVD” or “mitochondrial biogenesis” or “TFAM” or “PHF20” or “NRF1” or “PGC-1a”.

Search strategy used for systematic review and meta-analysis, including databases searched, key phrases, supplemental terms, and example search query. Searches were conducted as detailed in the Methods section of the main manuscript. Outlined is the full search used in PubMed; a similar strategy was adapted for CINAHL Ultimate.
