# Supplementary material for: Markers of clinical and mitochondrial adaptation in response to moderate intensity continuous training: A systematic review and meta-analysis
Source: PLoS One. 2026 Jan 2;21(1):e0339902. doi: 10.1371/journal.pone.0339902 (PMC12758752; doi:10.1371/journal.pone.0339902)
Supplement: S2 Table — Risk of Bias assessment for non-randomized studies of interventions (ROBINS-I). (DOCX) [file pone.0339902.s002.docx]

**Supplemental Table 2. Risk of Bias (ROBINS-I)**


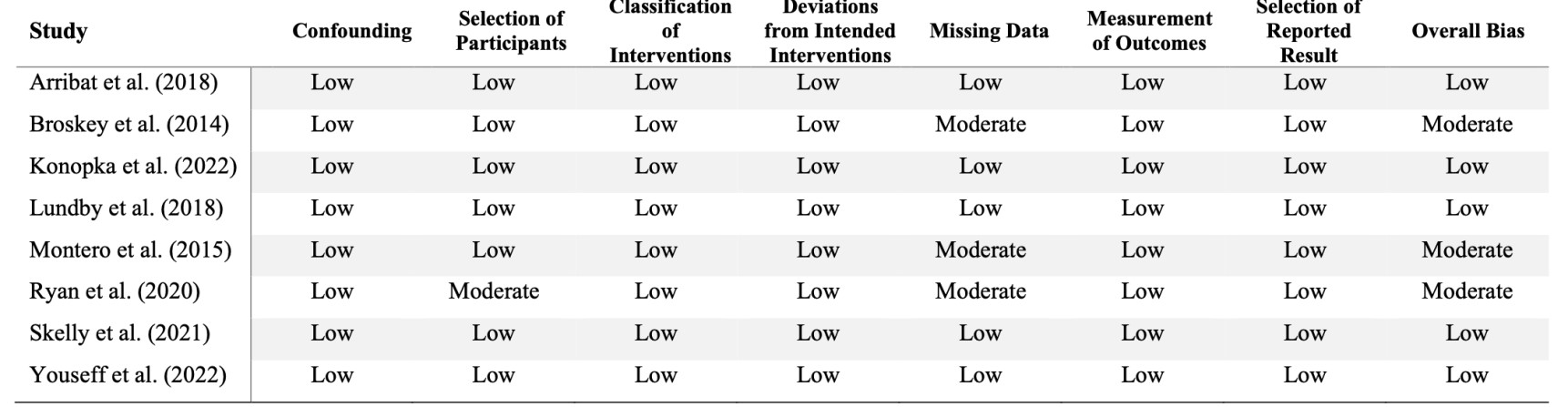


Risk of Bias assessment for non-randomized studies of interventions (ROBINS-I).
