# Supplementary figures and images for: Markers of clinical and mitochondrial adaptation in response to moderate intensity continuous training: A systematic review and meta-analysis
Source: PLoS One. 2026 Jan 2;21(1):e0339902. doi: 10.1371/journal.pone.0339902 (PMC12758752; doi:10.1371/journal.pone.0339902)

**Supplemental Table 3. Risk of Bias (ROB-2)**


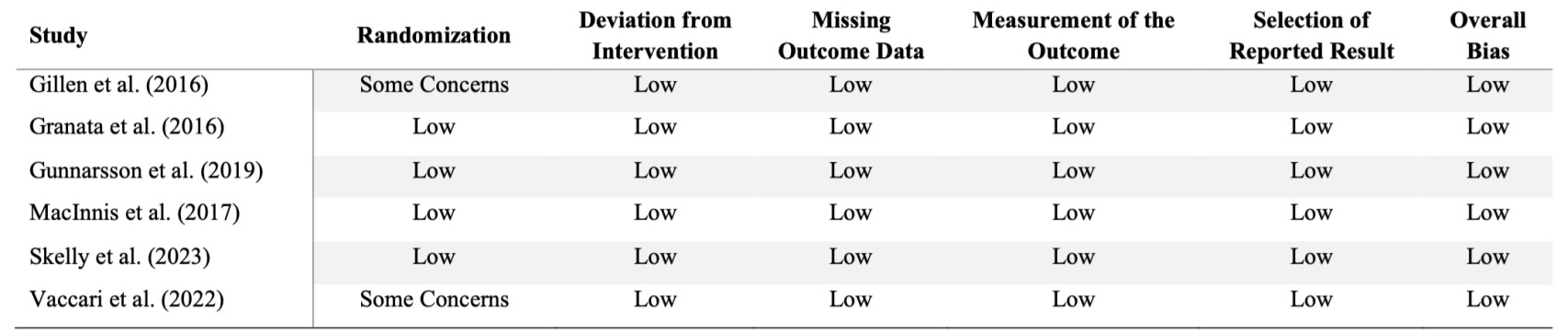


Risk of Bias assessment for randomized trials (ROB-2).

Supplement: S3 Table — Risk of Bias assessment for randomized trials (ROB-2). (DOCX) [file pone.0339902.s003.docx]
